# Nutrigenomic effect of conjugated linoleic acid on growth and meat quality indices of growing rabbit

**DOI:** 10.1371/journal.pone.0222404

**Published:** 2019-10-10

**Authors:** A. M. Abdelatty, Shereen A. Mohamed, Mahmoud M. A. Moustafa, Asmaa K. Al-Mokaddem, M. R. Baker, Ahmed A. Elolimy, Shawky A. Elmedany, Shaymaa Hussein, Omar A. A. Farid, Osama G. Sakr, Mohamed A. Elhady, Massimo Bionaz

**Affiliations:** 1 Department of Nutrition and Clinical Nutrition, Faculty of Veterinary Medicine, Cairo University, Giza, Egypt; 2 Department of Genetics and Genetic Engineering, Faculty of Agriculture, Benha University, Qalyubia, Egypt; 3 Department of Pathology, Faculty of Veterinary Medicine, Cairo University, Giza,Egypt; 4 Department of Physiology, Faculty of Veterinary Medicine, Cairo University, Giza, Egypt; 5 Department of Animal Sciences, University of Illinois, Urbana, IL, United States of America; 6 Regional Center for Food and Feed, Agriculture Research Center; Giza, Egypt; 7 Department of Cytology and Histology, Faculty of Veterinary Medicine, Cairo University, Giza, Egypt; 8 Department of Physiology, National Organization for Drug Control and Research, Giza, Egypt; 9 Animal Production Department (Poultry Physiology), Faculty of Agriculture, Cairo University, Giza, Egypt; 10 Department of Toxicology and Forensic Medicine, Faculty of Veterinary Medicine, Cairo University, Giza, Egypt; 11 Department of Animal and Rangeland sciences, Oregon State University, Oregon, United States of America; University of Agricultural Sciences, INDIA

## Abstract

Conjugated linoleic acid was detected in rabbit caecotrophs, due to the presence of microbial lipid activity in rabbit cecum. However, the effect of CLA as a functional food in growing rabbit is not well established. Therefore, this study was conducted to determine the effect of CLA on production, meat quality, and its nutrigenomic effect on edible parts of rabbit carcass including skeletal muscle, liver, and adipose tissue. Therefore, seventy five weaned V-Line male rabbits, 30 days old, were randomly allocated into three dietary treatments receiving either basal control diet, diet supplemented with 0.5% (CLAL), or 1% CLA (CLAH). Total experimental period (63 d) was segmented into 7 days adaptation and 56 days experimental period. Dietary supplementation of CLA did not alter growth performance, however, the fat percentage of *longissimus lumborum* muscle was decreased, with an increase in protein and polyunsaturated fatty acids (PUFA) percentage. Saturated fatty acids (SFA) and mono unsaturated fatty acids (MUFA) were not increased in CLA treated groups. There was tissue specific sensing of CLA, since subcutaneous adipose tissue gene expression of *PPARA* was downregulated, however, *CPT1A* tended to be upregulated in liver of CLAL group only (*P* = 0.09). In skeletal muscle, *FASN* and *PPARG* were upregulated in CLAH group only (*P* ≤0.01). Marked cytoplasmic vacuolation was noticed in liver of CLAH group without altering hepatocyte structure. Adipocyte size was decreased in CLA fed groups, in a dose dependent manner *(P <0*.*01)*. Cell proliferation determined by PCNA was lower *(P <0*.*01)* in adipose tissue of CLA groups. Our data indicate that dietary supplementation of CLA *(c*9,*t*11-CLA and *t*10,*c*12- CLA) at a dose of 0.5% in growing rabbit diet produce rabbit meat rich in PUFA and lower fat % without altering growth performance and hepatocyte structure.

## Introduction

In ruminants, the effect of microbial fermentation on dietary long-chain fatty acids (**LCFA**), especially biohydrogenation of polyunsaturated LCFA (**PUFA**), is relatively well known. The effect of microbial fermentation on PUFA in the hindgut of small herbivores including rabbit is poorly known [1,2]. For most non-ruminant herbivores this effect is likely irrelevant, considering that no LCFA present in the hindgut is absorbed; however, the coprophagy in rabbit let the LCFA that went through the hindgut microbial fermentation to be absorbed, influencing the fatty acid profiling in the body [2,3]. This could explain the presence of traces of *c9*,*t11*-C18:2 conjugated linoleic acid (**CLA**) in rabbit meat and adipose tissue [2,4], however, CLA (*c*9, *t*11-CLA) was only detected in neutral and not the polar lipid fraction of rabbit skeletal muscle [4], therefore, supplementation of CLA in rabbit feed is needed to study its nutrigenomic properties.

In several studies, supplementation or infusion of various CLA isomers has biological effects in ruminants. In dairy cows, milk fat content was decreased when rumen protected *t10*,*c12*-C18:2 CLA was infused in the abomasum; however, infusing *c9*,*t11*-C18:2 CLA did not affect milk fat [5]. The effect is not always observed, as duodenal infusion of *t10*,*c12*-C18:2 CLA did not decrease milk fat in dairy goats [6]. In non-ruminant animals, dietary supplementation of CLA affected growth performance, fatty acid profile, and meat quality [7–9].

The CLA, especially *t10*,*c12*-CLA, is known to have positive effect on human health [10,11]. It is not fully clear the mechanism for such effect, but CLA is considered a functional food, especially for its role on expression of genes via a nutrient-gene interaction (i.e., nutrigenomics). Especially important for the response to CLA are the peroxisome proliferator-activated receptors (**PPARs**) [12]. These nuclear receptors can strongly affect metabolism, especially lipid and glucose metabolism, with especially important role in muscle, liver, and adipose tissue [13–15]. Thus, the presence of traces of CLA in rabbit meat is of interest, however, the extent to which dietary suplementation of CLA to growing rabbit could affect the rabbit meat quality as a functional food, specifically affecting the transcriptome is not well known.

Based on the above, it seems reasonable to enrich rabbit meat with CLA through dietary supplementation of CLA.

Former studies manipulating the dietary supplementation of CLA (*c*9,*t*11-CLA and *t*10,*c*12- CLA) in growing rabbit are limited [16,17]. Evenmore, these studies did not investigate the effect of CLA at molecular level. This appears to be essential, considering the bioactivity of CLA in animal cells, especially due to its nutrigenomic property.

In order to close that gap, we aim in the present study to investigate the nutrigenomic, biological, and production effects of isomer mixture of *c*9,*t*11-CLA and *t*10,*c*12- CLA in weaned growing rabbit. We hypothesize that CLA affects meat quality and nutritional composition via affecting expression of lipid-related genes. Thus, the objective of this study was to assess the dose effect of equal proportion of two commonly used CLA isomers (*c9*,*t11*-CLA and *t10*,*c12*-CLA) on meat quality, production, and expression of lipid related genes in liver, muscle, and adipose tissue of weaned growing rabbit.

## Materials and methods

### Ethical approval

The experiment procedures for this study were approved by Cairo University Institutional Animal Care and Use Committee (approval# CU/II/F/47/17). And rabbits were euthanized according to AVMA (American Veterinary Medical Association) [18].

### Rabbits, housing, and diet

Seventy-five weaned white male V-Line rabbits 30 days old, were weighed and used in a 63 days experiment (7 days adaptation at which all rabbits were fed basal control diet; to adapt the animals to the new cage and management) and 56 days experimental period. Rabbits were housed individually under 12 hr light and 12 hr dark at a constant temperature (32±2°C). Rabbits had free access to water via nipple system.

The diets were formulated to meet or exceed the nutrient requirments recommended by NRC (National research council) for growing rabbits; 2500 Kcal digestible energy/ Kg diet, 16% crude protein, fat 2%, and crude fiber 10–12% [19] (**Table 1**). Rabbits were blocked for body weight (612.8 ± 24.89 g) and completely randomized into three (*n* = 25/group) isonitrogenous, isocaloric dietary treatments: 1) **CON** group was fed a basal control diet supplemented with 1% oleic acid (Techno Pharmachem, India), 2) **CLAL** group was fed a diet supplemented with 0.5% isomer-mix CLA (Lutrell Pure; BASF, Ludwigshafen,Germany; certified to contain equal proportion of *c*9,*t*11- and *t*10,*c*12-CLA; EFSA, [20, 21] plus 0.5% oleic acid, and 3) **CLAH** group was fed a diet supplemented with 1% isomer-mix CLA. Oleic acid was added to CON and CLAL diets to have final added lipid source of 1% according to a prior study [22]. All rabbits were fed basal control diet during the first week of the experiment followed by the CLAL and CLAH treated diets.

**Table 1:** Diet ingredients.

Ingredient	Diet %^1^
Barley	23
Soybean meal (44%)	16
Clover hay	30
Wheat Bran	26.3
Molasses	3
Lime stone	1
Sodium Chloride	0.4
Premix^2^	0.3
Total	100

^1^diet was formulated to provide 2500 Kcal digestible energy/ Kg diet according to rabbit NRC,1977

^2^The premix provides the following (per Kg diet): 15K IU of Vit.A; 100 mg Vit. E; 21mg Vit. K3; 10mg Vit B_1_; 40mg Vit. B_2_; 15mg Vit.B_6_; 0.1mg Vit.B_12_; 200mg Niacin; 100mg Pantothenic acid; o.5mg Biotin; 10mg Folic acid; 500mg Cholein Chloride; 450mg Zn; 600mg Mn; 0.3mg Fe; 50mg Cu; 250 mg I.

Based on our search we could not find a study on nutrigenomic effect of CLA on rabbits. Additionally, the few studies investigating the effect of CLA on rabbit only considered few parameters including meat fatty acids shift, meat quality, body weight. Those studies used dietary CLA at doses starting from 0.25% to 0.75% [16,17]. Effects on muscle fat and fatty acid composition were noticed at doses of 0.5% and 0.75%, respectively; therefore, we used 0.5% and 1% to induce changes on the gene level.

Feed samples were collected from each dietary treatment and analyzed (**Table 2**) for chemical composition according to AOAC, 2000 [23].

**Table 2:** Chemical composition of the experimental diets (DM%).

Item (%)	CON^1^	CLAL^2^	CLAH^3^
Dry matter	91.17	91.50	91.59
Crude protein	18.62	18.38	18.33
Crude fat	4.34	4.13	4.10
Ash	13.52	13.00	12.94
Crude fiber	15.44	14.75	14.55

^1^Control diet supplemented with 1% Oleic acid

^2^Diet with low dose of CLA contains 0.5% CLA+0.5% Oleic acid

^3^Diet with high dose of CLA contains 1% CLA

### Growth performance

All rabbits were individually weighed on weekly basis. Rabbits were individually fed, and feed intake was recorded weekly. Daily feed intake (DFI) on as-fed basis, average daily gain, and feed conversion ratio (FCR) were calculated.

### Euthanasia and blood sampling

At the end of the experimental period (d56), six rabbits were selected from each group (initial body weight of rabbits selected for euthanasia were non-significantly different between groups; P > 0.1) and used for all the analyses performed in this study. Rabbits were fasted overnight and euthanized according to AVMA guide lines [18], first, rabbits were anaesthetized according to a prior report [24] by intravenous injection of Ketamine and Xylazine (10 mg/kg, 3 mg/kg; respectively), followed by exsanguination. Blood samples were collected on vacutainer plain blood collection tubes (Lab supply, Cairo) and left for 30 min at room temperature before centrifugation at 1000×g for 15 min. Serum was stored in aliquots at -20°C. Serum was analyzed for total cholesterol (TC) and HDL-lipoprotein that regulates reverse cholesterol transport (RCT) to the liver and might explain the increased lipid accumulation in the liver of CLA (*c*9,*t*11-CLA and *t*10,*c*12- CLA) fed rabbits. HDL cholesterol was determined by enzymatic spectrophotometry according to manufacturer’s protocol (Spinreact, S.A./S.A.U.Ctra.Santa Coloma, Sant Estevede Bas, Spain). LDL cholesterol was calculated using the Friedewald formula: LDL = TC–HDL–(TG/5) [25].

The level of VLDL cholesterol was calculated according to the formula: VLDL = TG/5 [25,26]. Total cholesterol was determined according to the method of [27], and serum triglycerides (TG) were determined according to [28].

### Cecal pH and volatile fatty acids

After euthanasia, caecum was immediately exposed and pH was measured by pH probe (pH600, Milwakuee, Hungary). Caecal content (2–4 g) was collected in polypropylene tube and stored at -20°C for volatile fatty acids (VFAs) analysis using High Performance Liquid Chromatography (HPLC) according to [29]. The column was a Supelcosil C18 (5 μm particle and 80 A^o^ pore size) (250 x 4.6 mm), the mobile phase was 1% phosphoric acid with a flow rate of 2 ml/min. Compounds were detected at 210 nm wavelength. Chromatogram and data were captured using Agilent ChemStation software (Agilent Technologies, INC., USA).

### Carcass traits, meat composition, and fatty acid profile

After evisceration, weight of head, liver, kidney, heart, spleen, and hot carcass was recorded. The carcass was then cut into two equal longitudinal portions, the left side of the carcass was segmented to record the weight of mid quarter, fore, and hind limb. The right carcass was used for sampling, *Longissimus lumborum* muscle was excised and stored at -20°C for meat composition and determination of fatty acids content.

Meat composition analysis was carried out according to AOAC [23] and meat fatty acid profile was determined according to AOAC [30], by using gas chromatography (Shimadzu- 2010 plus; Shimadzu Co. Japan) equipped with flame ionization detector (FID-2010 PLUS, Shimadzu, Japan). Separation of fatty acid methyl esters (FAME) was carried out using Agilent capillary column (60m×0.25mm I.D.; Agilent, USA) coated with a BD-Wax (Agilent J&W, CA, USA) stationary phase. Injector temperature was 260°C, detector temperature was 260°C, and column temperature was 200°C. The run was 80 minutes. The individual FAME was identified by reference to the retention time of authentic FAME standard (Sigma Aldrich, USA). The relative proportion of each fatty acid in the sample was expressed as a percentage of the total fatty acids measured and calculated with the GC software.

### Histomorphometry and immunohistochemistry

#### Tissue samples

Tissue samples of liver, adipose tissue, and *Longissimus lumborum* muscle were collected and preserved in 10% neutral buffered formalin, processed and stained with hematoxylin and eosin according to [31].

Three slides were prepared and examined for liver, skeletal muscle, and subcutaneous adipose tissue (9 slides / rabbit; 6 rabbits per group).

### Histological examination and histometry

Tissue sections stained with hematoxylin and eosin were examined using Olympus BX43 light microscope and captured using Olympus DP27 camera linked to CellSens dimensions software (Olympus, Egypt). For liver lesions, histological scoring was done according to [32]. Briefly, for each rabbit sample, random fields (3–4 field) were evaluated for congestion and hepatocellular changes (Fatty change and intracellular edema) as follow: none = 0, zone III = 1, zone II, III = 2, Zone I, II, III (pan-acinar). Necrosis was evaluated as follow: [none = 0, single cell or focal (zone I) = 1, sub massive (bridging necrosis) = 2, bridging necrosis+ sub massive necrosis+ infarction = 3]. Total histopathological score was obtained by summation of the three scores. For histometry, three slides were prepared for each animal sample, then five microscopic fields (200X) were captured from each slide, the area (μm^2^) of adipocytes was determined, the mean of adipocyte size per group was then calculated and the number of adipocytes per area was also determined [33].

#### Immunohistochemical staining

Formalin fixed paraffin embedded adipose tissue sections were immunostained using proliferating cell nuclear antigen (**PCNA**) according to a prior study [34]. The PCNA-immunoreactive nuclei were counted and expressed as percentage per mm^2^ of tissue according to a prior report [35].

#### Transmission electron microscopy

Blocks of liver tissue (1-mm^3^) were immediately preserved in 4% cold gluteraldehyde after euthanasia and kept for transmission electron microscopy (TEM) at 4°C. Samples were then processed and embedded in Epon (embedding media; Sigma-Aldrich, Egypt). Semi-thin sections were stained with Toluidine blue and examined. Ultrathin sections were cut on an LKB Ultrotome III (LKB, Egypt), and photographs were taken with JEOL JEM-100CX II Electron Microscope (JEOL Ltd., Tokyo Japan) [36].

### Transcriptomic analysis

#### Extraction of RNA

Samples from liver, right *longissimus lumborum* muscle, and right supra neck fat lobe were collected, snap frozen in liquid nitrogen, and stored in -80°C till analysis. One gram of each tissue was ground to a homogenous powder using a mortar and pestle under liquid nitrogen [37] and used for total RNA extraction using Qiagen RNeasy mini-kits with on-column DNase digestion according to manufacturer’s instructions (Qiagen, Valencia, CA). RNA was collected in 30 μL of RNase free water and stored at -80 ᴼC. The RNA concentration and purity were measured by Nano-Drop 2000C spectrophotometer (Thermo Scientific, USA). The purity (absorbance ratio, A260/A280) was 2.00±0.10 for all samples. The integrity of RNA was verified on 2% agarose gel using gel electrophoresis image (Gel Doc. BioRad) according to [37].

#### Synthesis of cDNA template

For the cDNA synthesis, 300 ng of RNA per 20 ul reaction were used, cDNA was synthesized using SensiFast cDNA synthesis kits (Sigma Bioline, UK) according to the manufacturer’s instruction.

#### Primer designing and quantitative PCR

To investigate the effect of CLA (*c*9,*t*11-CLA and *t*10,*c*12- CLA) on different lipid metabolism processes, lipogenesis and oxidation, the following target genes were selected: *ACACA* (Acetyl-CoA Carboxylase Alpha), encodes for a rate limiting enzyme in fatty acid synthesis [38]; *CPT1A* (Carnitine Palmitoyl Tranferase 1 alpha), essential for the transport of long-chain fatty acids into the mitochondria for oxidation [39]; *FASN* (Fatty Acid Synthase) catalyzes de novo long-chain fatty acid synthesis [40]; *PPARG*,transcriptional regulation of adipocyte differentiation [41]; and *PPARA*, nutrient sensing and regulating the rate of fatty acid lipogenesis and catabolism in response to feeding [42].

Primer pairs for selected target and reference genes are summarized in **Table 3.** Primer pairs not previously published were designed using Primer Express 3, as previously described [43]. Primer pairs were purchased from (Genwez, USA). Desalted lyophilized primers were reconstituted using RNase free water (Invitrogen, USA).

**Table 3:** Gene names, primer sequences, accession#, and product size of the used genes.

Gene^1^	Accession #	Primers sequences(5’→3’)	Product size(bp)	Reference
*UXT*	XM_008272555	F: GCGGGACTTGCGAAAGGT	100	Designed
		R: AGCTTCCTGGAGTCGTTCAATG		
*RPS15A*	XM_008257787	F: AGCATGGTTACATTGGCGAGTT	100	Designed
		R:CTGATCACTCCGCACTTGTTTAA		
*MRPL39*	XM_008267147	F:CCTGTTCACTTACGAGCACATTTT	100	Designed
		R: TTGCATTCTGAAGAGATGTATCTATGG		
*PPARA*	XM_002723354	F:AGGCCCTCTTCAGAACCTGT	122	Lie et al., 2017
		R:GTGGCTTTCTGTTCCCAGAG		
*PPARG*	NM_001082148.1	F:GGAGCAGAGCAAAGAAGTCG	111	Lie et al., 2017
		R:CTCACAAAGCCAGGGATGTT		
*CPT1A*	XM_002724092.2	F:ATTCTCACCGCTTTGGGAGG	196	Lie et al., 2017
		R:ACGGGGTTTTCTAGGAGCAC		
*FASN*	KF201292.1	F: ACCACGTCCAAGGAGAGCA	112	Lie et al., 2017
		R: AGTTCTGCACCGAGTTGAGC		
*ACACA*	XM_002719077.2	F:GTGGTCTTCGTGTGAACTGG	122	Lie et al., 2017
		R:TTCTTCTGCTGCCTTTAGCC		

^1^UXT, ubiquitously-expressed, prefoldin-like chaperone; RPS15A, Ribosomal Protein S15a; MRPL39, mitochondrial large ribosomal subunit protein 39; PPARA, Peroxisome Proliferator Activated Receptor Alpha; PPARG, Peroxisome Proliferator Activated Receptor gamma; CPT1A, Carnitine Palmitoyl transferase 1A; FASN, Fatty Acid Synthase; ACACA, Acetyl-CoA Carboxylase Alpha.

The cDNA was diluted 1:20 using molecular grade RNase free water (Invitrogen, USA). Quantitative PCR reaction was performed using Maxima SYBR Green/ROX qPCR master mix (2x) (Thermo Scientific, USA). The reaction was performed in 0.2 ml qPCR strip tubes with optical caps (Gunster Biotech Co., Taiwan). The reaction consisted of 12.5μL of SYBR green Master Mix, 11 μL of diluted cDNA, and 0.75 μL of each forward and reverse primer pair to reach final volume of 25μL. Each sample was performed in triplicate. A non-template control (NTC) was run to check for primer dimer and RNA contamination. The reaction was performed in AriaMx Real Time PCR (Agilent Technologies, USA) using a two steps protocol: initial denaturation at 95°C for 10 min, then 40 cycles of denaturation at 95°C for 15 Sec followed by annealing/extension at 60°C for 60 seconds. A melting curve protocol was run at the end of the PCR by heating at 95°C for 30 Sec followed by a 65°C for 30 Sec, and 95°C for 30 Sec.

Fluorescent amplification curves data were downloaded from the AriaMx PCR System and analyzed using LinReg PCR software [44]. Three reference genes were tested including ubiquitously expressed transcript (*UXT*), small ribosomal protein Sub unit 15 (*RPS15A*), and mitochondrial large ribosomal subunit protein 39 *(MRPL39)*. The best combination of reference genes was determined using NormFinder [45]. The stability value of the combination of the two best reference genes (*RPS15A* and *UXT*) was 0.241 for adipose tissue, 0.262 for liver, and 0.168 for muscle. The normalization factor was calculated as the geometrical mean of the two best reference genes and final data of target genes were obtained by dividing the RTqPCR values obtained by LinRegPCR to the normalization factor.

### Statistical analysis

Data outliers were checked using Proc Reg of SAS (v9.4). Data with studentized t >2.5 were removed. Data were statistically analyzed using GLM procedures of SAS (v9.4) with orthogonal contrasts to obtain linear and quadratic effects with treatment as main effect. For histopathological score data, an ANOVA non-parametric approach with Proc Npar1way of SAS was used. If an overall significance of the Cui-Square was detected a pair comparison test was performed. Statistical significance was declared with a *P* ≤0.05 and tendency when *P* >0.05 but *P* ≤0.10.

## Results

### Growth performance

Growth performance parameters are presented in **Table 4**. Dietary CLA supplementation did not affect growth performance.

**Table 4:** The effect of dietary CLA on growth performance parameters of growing rabbit^1^.

Item	CON^2^	CLAL^3^	CLAH^4^	SEM	L*	Q*
Initial weight, (g)	612.80	612.80	612.80	12.60	1.00	1.00
Final weight, (g)	1879.4	1903.9	1885.00	32.3	0.90	0.56
Daily body weight gain, (g)	22.66	23.40	22.88	0.59	0.79	0.38
Daily feed intake, (g)	86.14	87.83	87.75	2.03	0.52	0.69
FCR	3.86	3.83	3.93	0.11	0.65	0.66

^1^ Values are least square mean, *n* = 25 samples /group

^2^ group fed on basal diet supplemented with 1% Oleic acid

^3^ group fed on diet supplemented with 0.5% CLA and 0.5% oleic acid

^4^ group fed on basal diet supplemented with 1% CLA

*P-value for the linear (L) or quadratic (Q) effect of the CLA dose.

### Biochemical parameters

Serum lipid profile is summarized in **Table 5**. There was a dose dependent increase (*P* < 0.01) in TC (CLAL, 9.5%; CLAH, 16.7%). However, triglycerides and VLDL cholesterol were equally increased only in CLAH group by >15%. The addition of CLA increased HDL cholesterol (CLAL, 19.0%; CLAH, 21.1%) but did not affect LDL cholesterol.

**Table 5:** The effect of CLA on serum lipid profile of growing rabbits^1^.

Item	CON^2^	CLAL^3^	CLAH^4^		SEM	L*	Q*
Total cholesterol, (mg/dl)	45.90^c^	50.28^b^	53.55^a^	0.67		<0.01	0.51
Triglycerides, (mg/dl)	61.00^b^	64.33^b^	70.33^a^	1.34		<0.01	0.43
HDL, (mg/dl)	19.33^b^	23.00^a^	23.41^a^	1.05		0.02	0.22
LDL, (mg/dl)	14.40	14.35	16.07	1.03		0.26	0.49
VLDL,(mg/dl)	12.20^b^	12.93^b^	14.07^a^	0.27		<0.01	0.56
TC/HDL	2.40	2.22	2.28	0.10		0.27	0.34
LDL/HDL	0.76	0.65	0.69	0.08		0.35	0.43

^1^ Values are least square mean, *n* = 6 samples /group.

^2^ group fed on basal diet supplemented with 1% Oleic acid.

^3^ group fed on diet supplemented with 0.5% CLA and 0.5% oleic acid.

^4^ group fed on basal diet supplemented with 1% CLA.

Different superscript letters denote P<0.05 between treatments.

*P-value for the linear (L) or quadratic (Q) effect of the CLA dose.

### Caecum pH and Volatile fatty acids

The caecal major volatile fatty acids concentration and pH value are summarized in **Table 6**. Dietary CLA *(c*9,*t*11-CLA and *t*10,*c*12- CLA) tended to decrease Acetate (*P* = 0.1) and butyrate (*P* = 0.11) in CLAH group by 20.41 and 25.1 percentage, respectively. However, caecal pH was significantly (*P* <0.01) decreased by -8.8% in CLAH group compared to the other groups.

**Table 6:** The effect of dietary CLA on caecal volatile fatty acids and pH of growing rabbits^1^.

Item	CON^2^	CLAL^3^	CLAH^4^	SEM	L*	Q*
pH	6.36^a^	6.26^a^	5.80^b^	0.08	<0.01	0.10
Acetate,(mg/g)	48.83	47.40	38.86	4.15	0.10	0.50
Propionate,(mg/g)	30.45	33.12	31.02	1.35	0.77	0.20
Butyrate,(mg/g)	20.58	20.08	15.42	2.22	0.11	0.45

^1^ Values are least square mean, *n* = 6 samples /group

^2^ group fed on basal diet supplemented with 1% Oleic acid

^3^ group fed on diet supplemented with 0.5% CLA and 0.5% oleic acid

^4^ group fed on basal diet supplemented with 1% CLA

Different superscript letters denote P<0.05 between treatments

*P-value for the linear (L) or quadratic (Q) effect of the CLA dose.

### Carcass traits, meat composition and Fatty acids profile

The effect of dietary CLA (*c*9,*t*11-CLA and *t*10,*c*12- CLA) on carcass characteristics and organs weight is summarized in **Table 7**. The liver weight was significantly increased (*P* = 0.01) in CLAH group compared to the other groups. Dietary CLA did not affect dressing % and different carcass cuts.

**Table 7:** The effect of CLA on carcass traits as % of the hot carcass weight of growing rabbits^1^.

Item	CON^2^	CLAL^3^	CLAH^4^	SEM	L*	Q*
Dressing (%)	59.78	60.39	60.13	0.67	0.71	0.61
Fore limb	14.00	13.98	14.75	0.30	0.10	0.30
Hind limb	34.09	34.97	32.94	0.56	0.17	0.06
Mid quarter	40.99	40.33	41.74	0.66	0.43	0.22
Head	9.47	9.56	9.62	0.31	0.74	0.96
Liver	4.54^b^	4.31^b^	5.55^a^	0.24	0.01	0.02
Kidney	1.18	1.06	1.18	0.05	0.95	0.08
Heart	0.59	0.48	0.60	0.05	0.84	0.07
Spleen	0.11	0.09	0.09	0.01	0.44	0.71

^1^ Values are least square mean, *n* = 6 samples /group

^2^ group fed on basal diet supplemented with 1% Oleic acid

^3^ group fed on diet supplemented with 0.5% CLA and 0.5% oleic acid

^4^ group fed on basal diet supplemented with 1% CLA

Different superscript letters denote P<0.05 between treatments

*P-value for the linear (L) or quadratic (Q) effect of the CLA dose.

Meat composition and fatty acid profile of *Longissimus lumborum* muscle is shown in **Table 8**. Compared to control, the protein content of the *Longissimus lumborum* muscle increased by 5.1% and 10.3% (*P* = 0.01) in CLAL and CLAH groups, respectively. The fat composition decreased by almost the same percentage (-7.3 and—15.6%) in CLAL and CLAH groups, respectively.

**Table 8:** Chemical composition and fatty acid profile of *Longissimus lumborum* muscle of growing rabbit fed Control or CLA supplemented diet^1^.

Item	CON^2^	CLAL^3^		CLAH^4^	SEM		L*	Q*
Moisture,%	74.6^a^	73.9^b^	73.3^c^		0.15		<0.01	0.96
Protein,%	20.0^c^	21.0^b^	22.0^a^		0.12		<0.01	0.96
Fat,%	3.85^a^	3.57^b^	3.25^c^		0.08		<0.01	0.87
Ash,%	1.57	1.48	1.43		0.07		0.21	0.85
***Fatty Acids*)% of total fatty acids)**								
C14:0	1.89^a^	1.72^b^	1.60^b^		0.04	<0.01		0.60
C16:0	30.0^a^	27.6^b^	25.4^c^		0.23	<0.01		0.55
C16:1n7	1.40^a^	1.21^b^	1.00^c^		0.02	<0.01		0.75
C16:3n4	0.38^c^	0.41^b^	0.52^a^		0.01	<0.01		<0.01
C17:0	1.36	1.34	1.35		0.02	0.62		0.55
C18:0	9.07^b^	9.13^ab^	9.4^a^		0.09	0.02		0.38
C18:1n7	1.25^b^	1.30^b^	1.39^a^		0.02	<0.01		0.48
C18:1n9	27.9^a^	26.0^b^	24.2^c^		0.20	<0.01		0.81
C18:2n4	0.30	0.32	0.32		0.01	0.06		0.46
C18:2n6	21.3^c^	26.3^b^	29.7^a^		0.18	<0.01		<0.01
C18:3n6	0.30^b^	0.30^b^	0.52^a^		0.01	<0.01		<0.01
C18:3n3	2.38^a^	2.22^b^	2.04^c^		0.04	<0.01		0.91
C18;3n4	0.33^b^	0.36^a^	0.34^b^		0.01	0.71		<0.01
C18:4n3	0.37^a^	0.30^b^	0.30^b^		0.01	<0.01		<0.01
C20:0	0.35^a^	0.31^b^	0.32^b^		0.01	0.05		0.12
C20:1n5	0.35^c^	0.38^b^	0.51^a^		0.01	<0.01		<0.01
C20:1n9	0.28	0.26	0.26		0.01	0.14		0.18
C22:0	0.28	0.30	0.31		0.01	0.08		0.88
Σ SFA^5^	43.0^a^	40.4^b^	38.4^c^		0.24	<0.01		0.26
Σ MUFA^6^	31.2^a^	29.2^b^	27.4^c^		0.20	<0.01		0.60
Σ PUFA^7^	25.4^c^	30.2^b^	33.7^a^		0.19	<0.01		0.01

^1^ Values are least square mean, *n* = 6 samples /group

^2^ group fed on basal diet supplemented with 1% Oleic acid

^3^ group fed on diet supplemented with 0.5% CLA and 0.5% oleic acid

^4^ group fed on basal diet supplemented with 1% CLA

^5^ Saturated fatty acids

^6^ Monounsaturated fatty acids

^7^Poly unsaturated fatty acids

Different superscript letters denote P<0.05 between treatments

*P-value for the linear (L) or quadratic (Q) effect of the CLA dose.

Supplementing rabbit diet with CLA (*c*9,*t*11-CLA and *t*10,*c*12- CLA) decreased the proportion of C16:0 and C18:1n9 in CLAL and CLAH while increased the proportion of C18:2n6 in a dose dependent manner (*P* < 0.01). Moreover, proportion of the C18:3n6 was increased (*P* < 0.01) by CLAH but not by CLAL. Proportion of total saturated fatty acids and mono-unsaturated fatty acids was negatively affected by CLA while % of PUFA was increased in a dose dependent manner by CLA (*P* < 0.01).

### Histopathological findings

#### Histological examination

**Fig 1:**
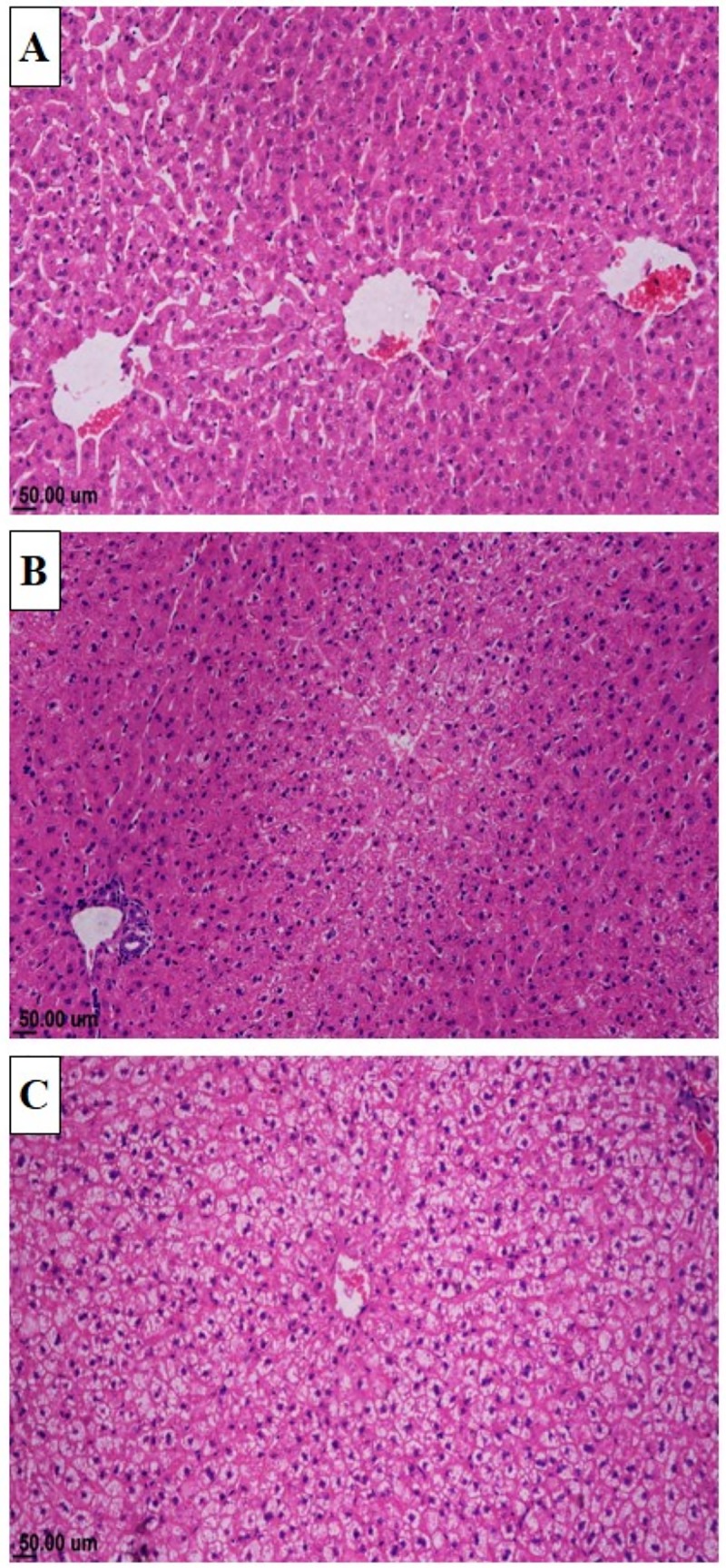
Microscopic view of liver of growing rabbits receiving normal or CLA supplemented diet. Photomicrograph of rabbit liver (a) Normal histological structure of hepatic parenchyma, control group (H&E), (b) Mild vacuolation of the hepatocytes at the centrilobular area, Group received 0.5% CLA (H&E) and (c) Marked vacuolar degeneration in the hepatocytes with presence of cytoplasmic shreds, Group received 1% CLA (H&E).

Histological examination of liver sections of rabbit from CON group revealed normal architecture of hepatic parenchyma **Fig 1A.** Liver sections from rabbit receiving CLA presented vacuolation of the hepatocytes cytoplasm (**Fig 1B and 1C**). The vacuolation was mild in CLAL (**Fig 1B**) but marked in CLAH (**Fig 1C**). Histological score of liver lesions is presented in **Table 9**. Total hepatic lesion of CLAH group was significantly higher than that of CON group (P ≤ 0.01).

**Table 9:** Histological scoring of liver^1^.

Parameter	CON^2^	CLAL^3^	CLAH^4^	P*
Congestion	0.70	0.90	1.20	0.26
Hepatocellular changes	0.50^c^	1.30^b^	2.50^a^	<0.01
Necrosis	0.30	0.40	0.80	0.19
Total	1.50^b^	2.60^b^	4.50^a^	<0.01

^1^ Values are mean of score as calculated SAS, *n* = 6

^2^ group fed on basal diet supplemented with 1% Oleic acid

^3^ group fed on diet supplemented with 0.5% CLA and 0.5% oleic acid

^4^ group fed on basal diet supplemented with 1% CLA

Different superscript letters denote P < 0.05 between treatments

*P value for One-Way analysis Chi-Square among treatments

**Fig 2:**
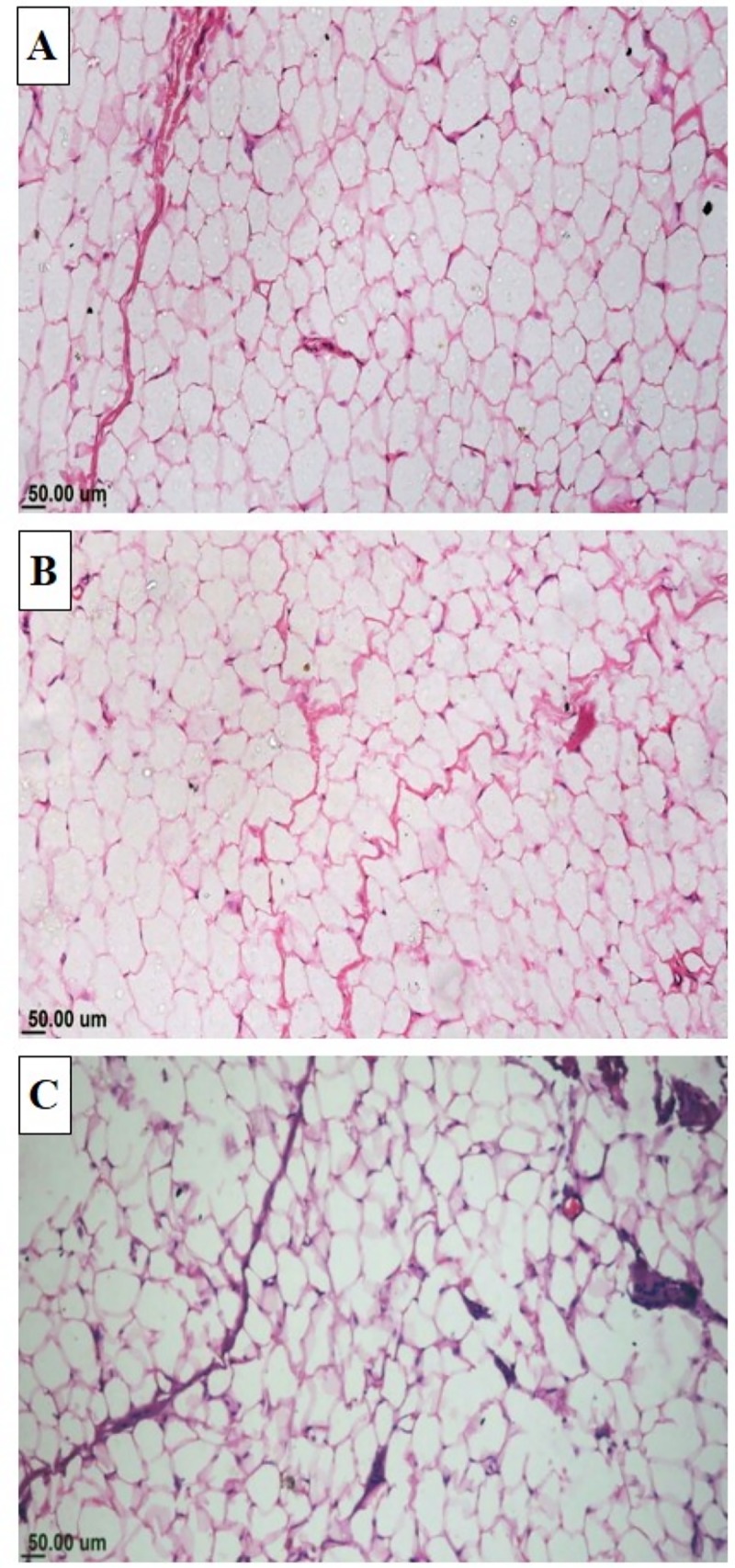
Microscopic view of subcutaneous fat of growing rabbits receiving normal or CLA supplemented diet. Photomicrograph of rabbit subcutaneous fat showing the normal structure of mature adipose tissue with presence of numerous monolocular adipocytes and scanty connective tissue septa (H&E). (a) Control group. (b) Group received 0.5% CLA and (c) Group received 1% CLA.

Adipose tissue of all groups revealed mature monolocular adipocytes with fine connective tissue septa, in-between that housed small blood capillaries **Fig 2**. Administration of CLA (*c*9,*t*11-CLA and *t*10,*c*12- CLA) resulted in significant decrease (P ≤ 0.05) in adipocyte size with increase (P ≤0.05) in the number of cells per unit area **(Table 10)**.

**Table 10:** Adipocyte area (μm^2^), Adipocyte number per unit area and percentage of PCNA positive cells^1^.

Parameter	CON^2^	CLAL^3^	CLAH^4^	SEM	L*	Q*
Adipocyte area (**μ**m^2^)	9712.3^a^	5319.4^b^	3793.4^c^	369.1	<0.01	<0.01
Adipocyte Number (n/area)	20.1^a^	25.1^b^	29.1^c^	0.5	<0.01	0.41
PCNA^5^ positive nuclei (%/mm^2^)	16.6^a^	10.1^b^	6.3^c^	0.5	<0.01	0.08

^1^ Values are least square mean, *n* = 6/group

^2^ group fed on basal diet supplemented with 1% Oleic acid

^3^ group fed on diet supplemented with 0.5% CLA and 0.5% oleic acid

^4^ group fed on basal diet supplemented with 1% CLA

^5^ Proliferating cell nuclear antigen

*P-value for the linear (L) or quadratic (Q) effect of the CLA dose.

#### Immunohistochemistry

**Fig 3:**
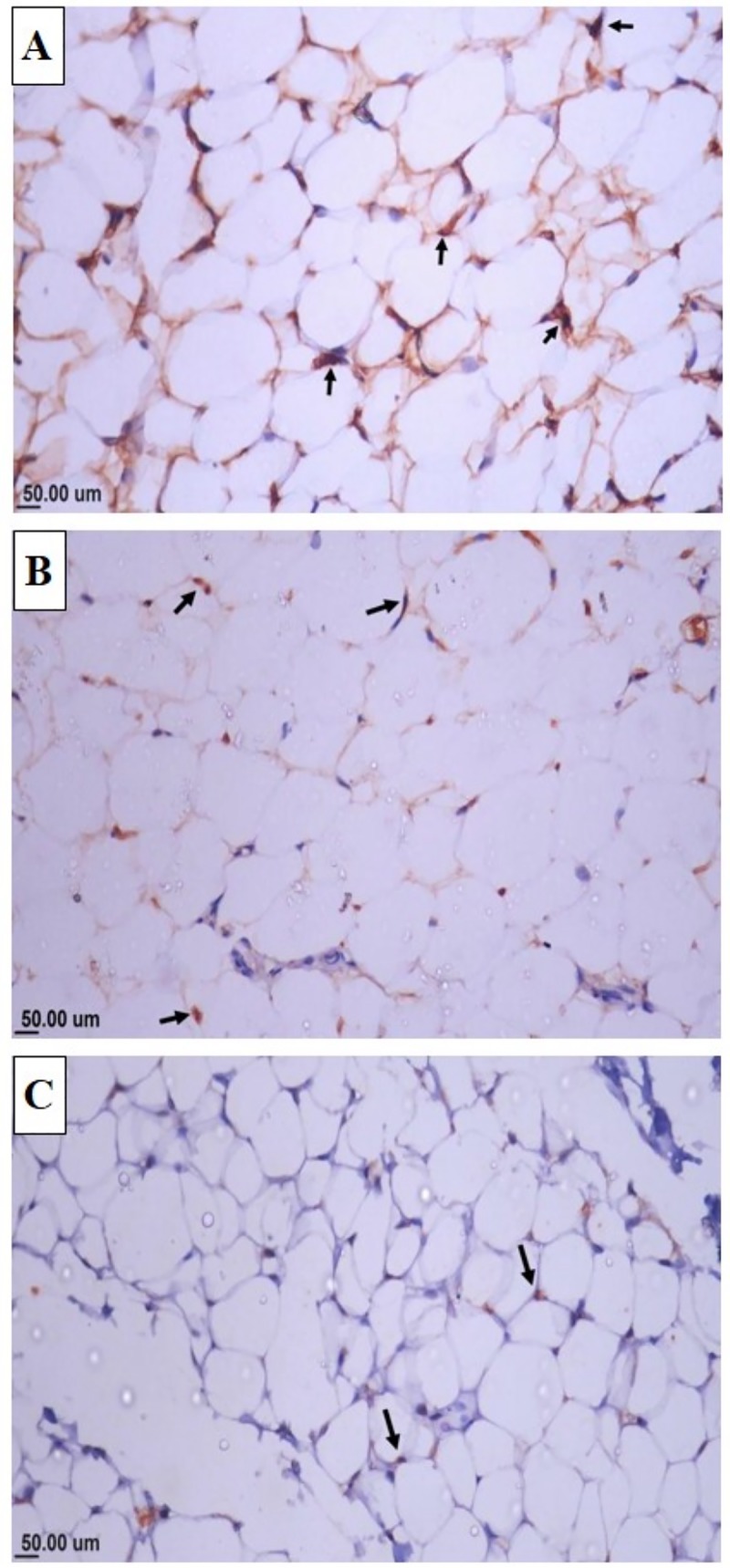
Microscopic view of adipose tissue immune-stained with PCNA of growing rabbits receiving normal or CLA supplemented diet. Photomicrograph of adipose tissue immune-stained with PCNA showing (a) marked expression of PCNA positive nuclei in control group (arrow), (b) mild expression of PCNA positive nuclei in CLAL group (arrow) and (c) scanty number of PCNA positive nuclei in CLAH group (arrow).

As shown in **Table 10** and **Fig 3**, the percentage of PCNA positive nuclei was significantly lower (P ≤0.05) in adipose tissue of CLAL and CLAH fed rabbits in a dose dependent manner (10.1 9.6% and 6.3 5.6%, respectively) when compared to control group receiving basal diet (17.3%), indicating the ability of CLA to decrease proliferation rate of the adipocytes.

#### Transmission electron microscopy Findings

**Fig 4:**
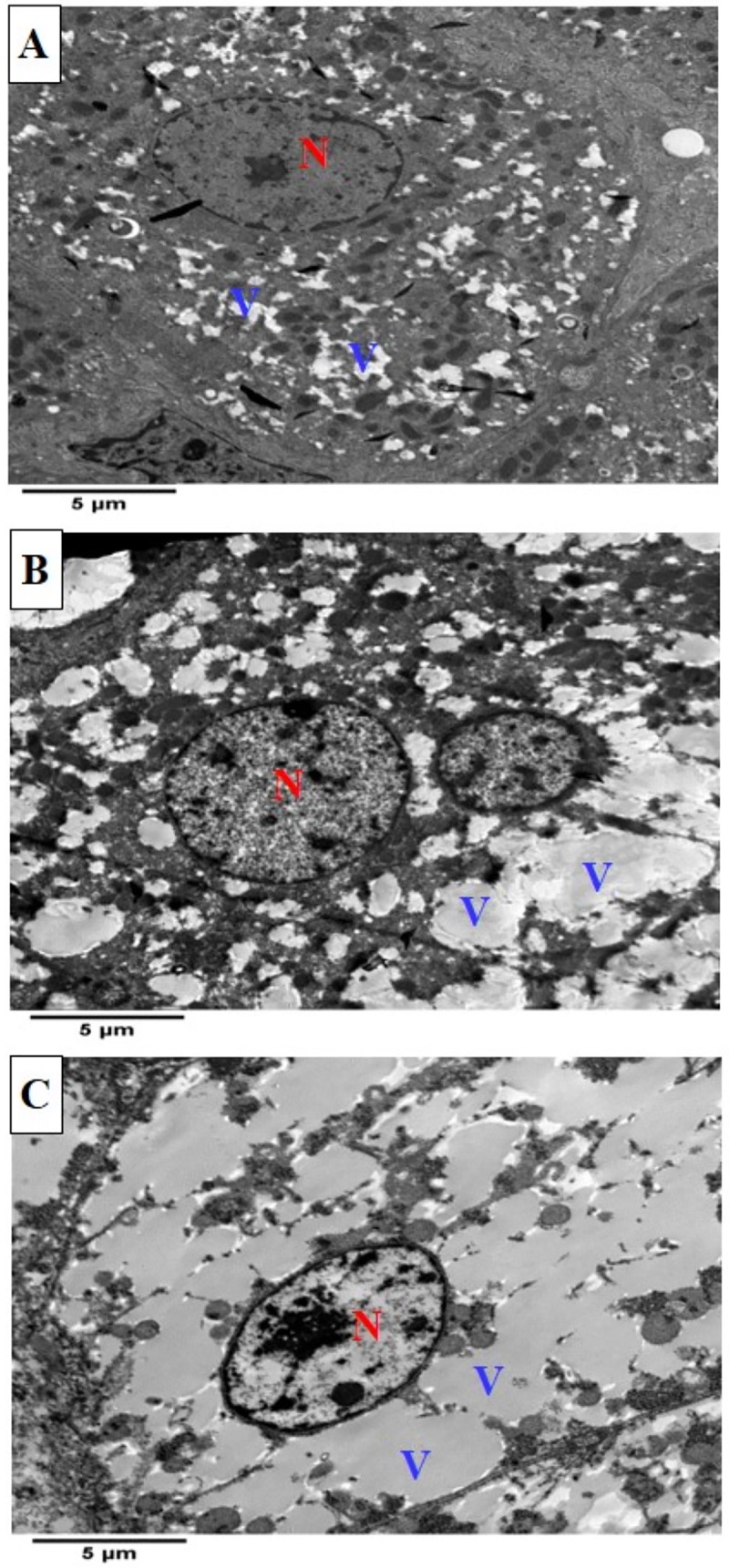
Transmission electron micrograph (TEM) of rabbit hepatocytes in the different experimental groups. As shown in TEM, hepatocyte of CON group (a), normal nucleus (N), fine small sized cytoplasmic vacuoles (V) dispersed within the cytoplasm, TEM of hepatocyte of CLAL group (b) showing few medium sized cytoplasmic vacuoles (V) and numerous small sizes ones, and TEM of hepatocyte of rabbit from CLAH group (c) showing numerous large cytoplasmic vacuoles (V).

As shown in transmission electron microscopy findings in **Fig 4**, Hepatocytes of all groups have shown normal hepatocyte structure, however, only hepatocytes of CLAH group exhibited large sized numerous cytoplasmic vacuoles while CLAL group showed few medium sized vacuoles, supporting the light microscopic findings.

**Fig 5:**
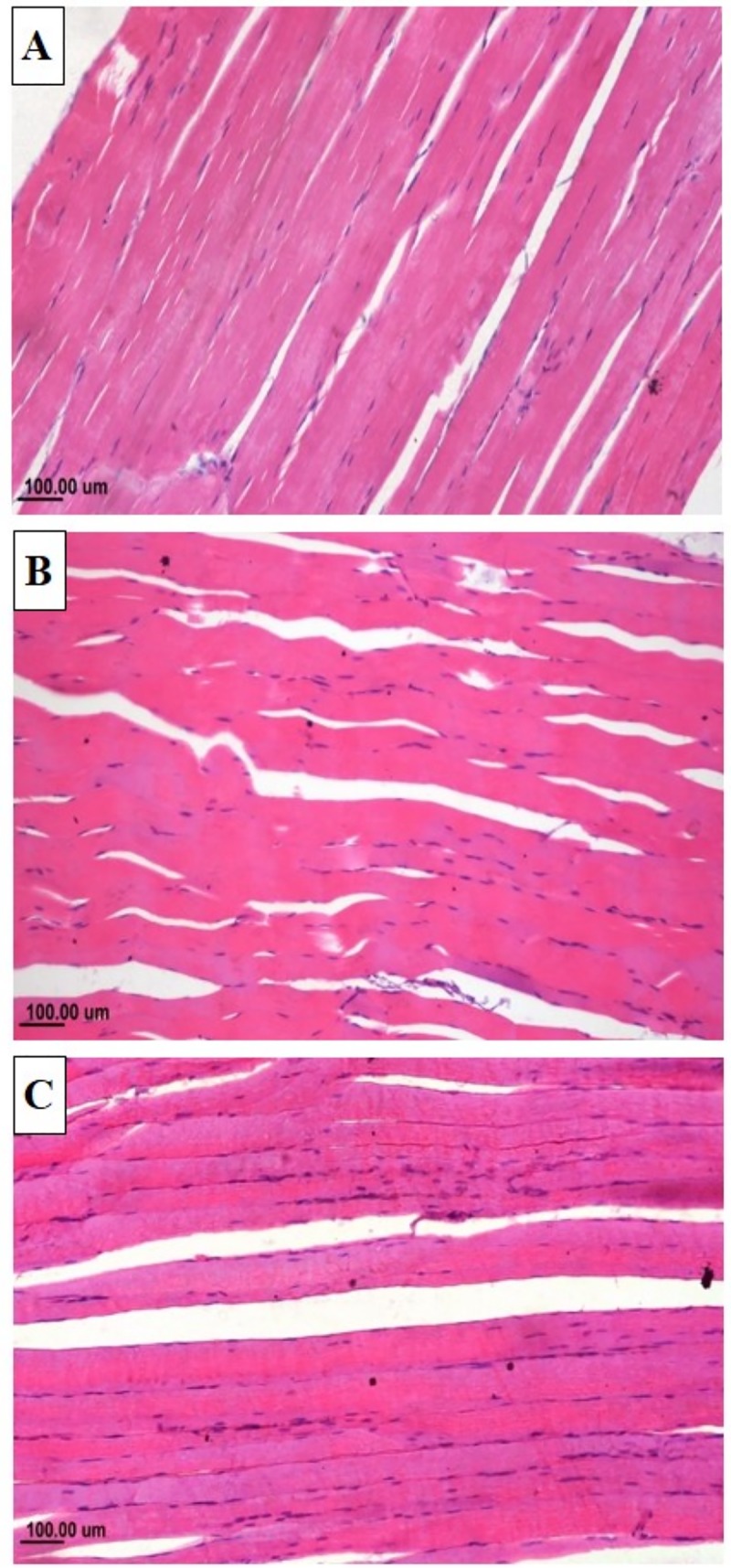
Effect of CLA (*cis*9, *trans* 11, and *trans* 10, *cis* 12-CLA) on skeletal muscle of growing rabbits. photomicrograph of skeletal muscle (longissimus lumborum) of rabbit, showing the normal histological structure of muscle bundles in (a) CON, (b) CLAL, and (c) CLAH groups.

Histological examination of *Longissimus lumborum* muscle revealed normal structure and arrangement of muscle bundles in all experimental groups (**Fig 5**), intact muscle bundles were arranged parallel to each other’s with elongated nuclei scattered peripherally under their sarcolemmal cover and visible cross striations.

### Liver, Muscle, and adipose gene expression

**Fig 6:**
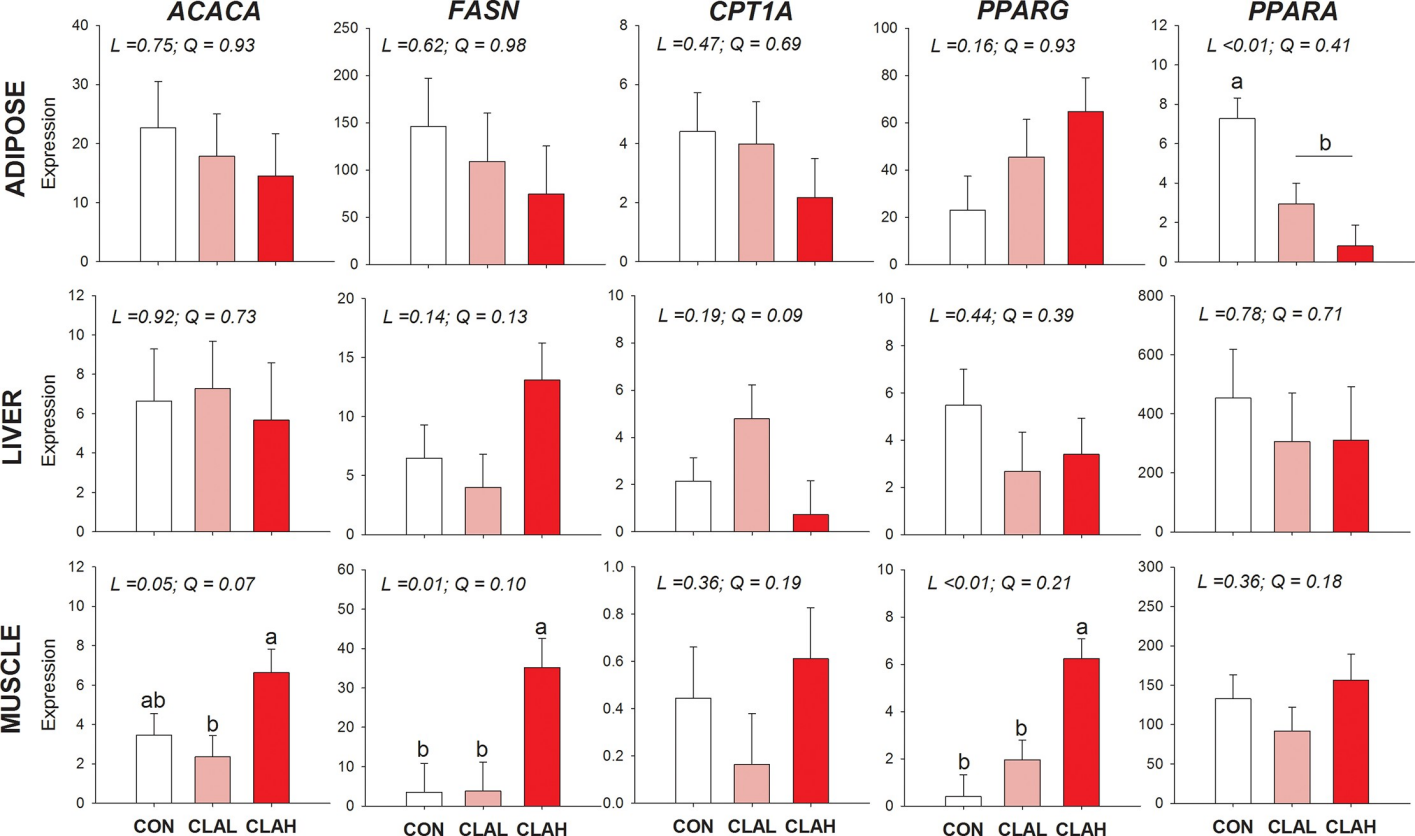
The effect of CLA supplementation on gene expression levels of genes regulating lipid metabolism in liver, skeletal muscle, and subcutaneous adipose tissue of growing rabbits. Growing rabbits were fed on either basal diet (CON) or diet supplemented with 0.5% CLA (CLAL) or 1% CLA (CLAH). The expression of *PPARA*, Peroxisome Proliferator Activated Receptor Alpha; *PPARG*, Peroxisome Proliferator Activated Receptor gamma; *CPT1A*, Carnitine Palmitoyl transferase 1A; *FASN*, Fatty Acid Synthase; *ACACA*, Acetyl-CoA Carboxylase Alpha were measured in liver, skeletal muscle, and adipose tissue, values are presented as LSM± SEM. Statistical significance was determined by ANOVA with Duncan's multiple-range test, with levels of different letters means significant difference.

The effect of dietary CLA (*c*9,*t*11-CLA and *t*10,*c*12- CLA) on the expression of genes regulating lipid metabolism in liver, *Longissimus lumborum* muscle, and subcutaneous adipose tissue is presented in **Fig 6**. Dietary supplementation of CLA in growing rabbit diets at 0.5% and 1% induced tissue specific changes in some major key genes involved in regulation of lipid metabolism. In adipose tissue, the dietary CLA supplementation downregulated the expression of *PPARA* in a dose dependent manner (*P* <0.01). There was a tendency for a linear effect of the dose of CLA to increase the expression of *PPARG* (*P* = 0.16).

In liver there was a numerical increase in transcription of *FASN* (*P* = 0.14) in CLAH group, and a numerical increase in expression of *CPT1A* by CLAL (*P* = 0.19). The effect of CLA on skeletal muscle gene expression was more prominent than liver due to an upregulation of *ACACA*, *FASN*, and *PPARG* by CLAH (P ≤ 0.05) compared to CON group.

## Discussion

Enriching rabbit meat with CLA can render rabbit meat a functional food for human consumption. However, the biological mechanism underlining the growth and meat quality modulation by CLA is not well studied in rabbit. Therefore, the present study investigated the effect of two doses (0.5 and 1%) of CLA isomer mix (*c*9,*t*11-CLA and *t*10,*c*12- CLA) on growth performance, meat composition, and expression of lipid related genes in liver, skeletal muscle, and subcutaneous fat which are major metabolic organs and edible carcass parts of growing rabbits.

Dietary supplementation of CLA (*c*9,*t*11-CLA and *t*10,*c*12- CLA) in growing rabbit diet did not alter growth performance parameters, which is similar to findings in a former study [46] on growing rabbit. However, the treatments tended to affect the fermentation in the hindgut, with significant decrease in pH, which likely has partly driven the numerical decrease levels of acetate and butyrate but did not affect propionate, these volatile fatty acids are the major end products of hind gut microbial fermentation [47], indicating an effect of CLA on gut microbiome. In a former study on mice, CLA was found to affect gut microbiota [48]. Therefore, our data are indicative of an effect of CLA (*c*9,*t*11-CLA and *t*10,*c*12- CLA) on microbiota, which warrant further studies.

Supplementation of CLA (*c*9,*t*11-CLA and *t*10,*c*12- CLA) increased total cholesterol that was driven by increase in HDL cholesterol in apparently healthy rabbits. These findings are in line with findings from [49–51]. On the other side, the dose dependent increase in TC in this study is in line with [52] and contrary to [16]; however, in their study[16], they supplied sunflower oil in the control diet. Although dietary supplementation of sunflower oil increased *c9*,*t11*-CLA in milk of dairy cow [53], that might indicate a confounding effect of using sunflower oil in the control diets in CLA studies.

In rodents, the response of plasma HDL to CLA is isomer specific, and is also not consistent, with reported increase in *t*10,*c*12-CLA fed rats [54] or no effect, in mice fed either *c*9,*t*11-CLA or *t*10,*c*12-CLA [55] on HDL cholesterol; however, in both studies serum triglycerides was decreased by both *c*9,*t*11-CLA or *t*10,*c*12-CLA. More similar to rodents, in our study HDL cholesterol was positively associated with CLA. Higher HDL cholesterol is associated in human with lower risk of atherosclerosis [56], it also did not increase the TC/HDL ratio similar to the control group, indicating the atherosclerosis protecting effect of CLA similar to [57]; however, high triglycerides is associated with increased risk of atherosclerosis [58]. In our study, CLA increased serum triglycerides. It is unclear the reason for such increase, but it could have caused the observed increase in lipid accumulation in liver. Despite, the presence of cytoplasmic vacuoles in hepatocyte due to lipid accumulation without affecting the hepatocyte structure as shown in TEM images in this study, indicate that CLA at the dose of 0.5% and 1% did not negatively affect hepatocyte structure. It is possible that CLA increased the cholesterol reverse transport by stimulating HDL production in the liver [59], indicating that CLA might stimulate the liver to produce more HDL when total cholesterol increases.

The effect of CLA on hepatocytes is multifactorial as previously observed in mice [60]. In the current study, the increased proportions of liver weight might be due to increase fat accumulation, as indicated by the presence of cytoplasmic vacuoles in hepatocytes in CLA supplemented rabbits [61]. The increase in the proportion of liver weight might be due to isomer specific action of *t10*,*c12*-CLA. An increase in liver weight was detected at a dose of 2% and 3% in hamsters [62]. The higher fat content in the liver in this study might be due to muliple factors including reduction of fatty acid oxidation rate in CLAH group due to decreased CPT1A. In addition to a numerical upregulation of *FASN* in CLAH, coding for a key *de-novo* fatty acid synthesis enzyme. Our data are similar to a prior study wherease, non-significant increase in hepatic FASN when 1.5% CLA was dietary supplemented to lactating mice [63]. Despite the fact that *PPARA* is more abundant with critical functions in liver than adipose tissue [64], recent studies revealed important functions of this genes in adipose tissue of rodents [65,66]. The latter studies might help to explain the effect of CLA on transcription of *PPARA* in adipose tissue rather than liver in the current study. Dietary CLA (c9,t11 and t10,c12- CLA) supplementation downregulated the expression of *PPARA* in sub-cutaneous adipose tissue in our study, similar to a prior study in mice [63]. Lower transcription of *PPARA* might be associated with lower lipid oxidation but also lower adipogenesis [66]. The decreased expression of PCNA positive cells indicating the ability of CLA to limit adipocytes proliferation was reported in a prior study [33], this reduction in proliferation rate could be attributed to the downregulation of *FASN*. Association between expression/function of *FASN* with adipocyte proliferation was reported in mice pre-adipocytes *in vitro* [67, 68].

In this study, there was a noticeable nutrigenomic effect of CLA on skeletal muscle besides an effect on composition and fatty acid profile. The increased level of PUFA (especially, C18:2n6 and C18:3n6) on the expense of MUFA and SFA by feeding CLA is consistent to prior studies [69,70] including rabbit [46]. The higher proportion of PUFA might be explained by the effect of CLA on steroyl CoA desaturase (*SCD*) gene expression that was increased in *longissimus thoracic* muscle of finishing pig [71]; however, expression of *SCD5* was not measured in the current study. In addition, dietary supplementation of CLA at a dose of 1% (CLAH) markedly upregulated skeletal muscle gene expression of lipid-synthesis associated genes, such as *FASN* and *PPARG* similar to prior studies [72,73]. Based on a prior study in beef steers [74], increase in expression of those two genes is associated with increased marbling. However, in our study the fat content in muscle was the lowest with CLAH as well as the fatty acid derived from de novo synthesis (C14:0 and a proportion of C16:0). Thus, the data appears contradictory and unexplained. It is possible that CLA affected muscle fat via effect on other genes or proteins, such as AMP-activated protein kinase, controlling lipolysis, and PPARδ [75–77]. In this context, the isomer specific action of CLA was reported by [78], they find that AMPK phosphorylation was increased by *t*10,*c*12 CLA and not *c*9,*t*11CLA.

The effect of different CLA isomers on crosstalk between liver, skeletal muscle, and adipose tissue in terms of regulation of lipid metabolism is not well understood, from our findings it seems that the effect of *c*9,*t*11-CLA or *t*10,*c*12- CLA on liver, sleletal muscle, and adipose tissue is not regulated through *PPARA*, similar to our findings is [79]. The Further investigations of different signalling pathways to undestand the regulatory effect of CLA on lipid metabolism in skeletal muscle-adipose tissue axis including AMPK, mTOR, and Glucocorticoid receptors need to be addressed.

## Conclusion

In this study we hypothesize that dietary supplementation of CLA mixture of *c9*,*t11*-CLA and *t10*,*c12*-CLA isomers in growing rabbit would improve rabbit meat quality via controlling expression of lipid-related genes without altering growth performance. Our findings confirmed that CLA mixture of c9,t11-CLA and t10,c12-CLA improved meat quality through increasing protein content and PUFA through a nutrigenomic action on lipogenic gene expression in skeletal muscle. The effect was present to a lesser extent in the liver with increased lipid accumulation without altering hepatocyte structure and with minimal effect on expression of measured genes. From our findings, dietary supplementation of CLA mixture of c9,t11-CLA and t10,c12-CLA to growing rabbit seems to have a tissue specific nutrient-gene interaction (muscle > adipose > liver), which needs further investigation by supplementing each isomer individually, and study its effects on genes regulating protein synthesis.
